# Patient-Reported Experiences with First-Time Naturopathic Care for Type 2 Diabetes

**DOI:** 10.1371/journal.pone.0048549

**Published:** 2012-11-07

**Authors:** Erica B. Oberg, Ryan Bradley, Clarissa Hsu, Karen J. Sherman, Sheryl Catz, Carlo Calabrese, Daniel C. Cherkin

**Affiliations:** 1 School of Naturopathic Medicine, Bastyr University, Kenmore, Washington, United States of America; 2 Group Health Research Institute, Seattle, Washington, United States of America; 3 Naturopathic Physicians Research Institute, Portland, Oregon, United States of America; College of Tropical Agriculture and Human Resources, University of Hawaii, United States of America

## Abstract

Differences in the effectiveness of diverse healthcare providers to promote health behavior change and successful diabetes self-care have received little attention. Because training in naturopathic medicine (NM) emphasizes a patient-centered approach, health promotion, and routine use of clinical counseling on wellness and prevention, naturopathic physicians (NDs) may be particularly well-prepared for promoting behavior change. However, patients’ experiences with NM have not been well studied. This study provides the first report of the perceptions of persons with type 2 diabetes of their first experiences with naturopathic care for their diabetes. Following their participation in a one-year prospective cohort study of adjunctive naturopathic care for diabetes, twenty-two patients were interviewed about their experiences working with a naturopathic physician. Using a content analysis approach, nine dominant themes were identified. Three themes characterized the nature of the ND-patient interaction: 1) patient-centered, 2) holistic health rather than diabetes focused, and 3) collaborative. Five themes characterized the content of the clinical encounter: 1) individualized and detailed health promotion, 2) counseling that promoted self-efficacy, 3) pragmatic and practical self-care recommendations, 4) novel treatment options that fostered hopefulness, and 5) patient education that addressed both diabetes self-care and general health. A ninth theme was cross-cutting: the contrast between ND care and conventional medical care. Results indicate that the routine clinical approach used by NDs is consistent with behavior change theory and clinical strategies found most effective in promoting self-efficacy and improving clinical outcomes.

## Introduction

Diabetes is a chronic disease requiring substantial patient engagement and self-management for successful control. Despite well-established clinical guidelines, most persons with diabetes struggle with managing their diet, physical activity, and glucose self-monitoring [1], [2]. Diabetes risk factors are modifiable with health behavior change, yet meaningful health promotion counseling is uncommon in primary care [3]–[5] and few physicians are extensively trained in the counseling techniques known to promote self-efficacy and support self-management [6], [7]. In conditions such as diabetes, where psychosocial barriers are common and challenging, and fostering self-efficacy and self-care is critical, identification of clinical strategies that successfully promote behavior change would be particularly valuable.

One type of health care provider, naturopathic physicians (NDs), who practice a broad spectrum of complementary and alternative medicine (CAM), routinely include clinical counseling on wellness and prevention and prioritize health promotion [8]–[11]. NDs treat a wide variety of health concerns similar in scope to those seen by conventional primary care [12]. NDs are currently licensed in 16 states, the District of Columbia, and the United States territories of Puerto Rico and the United States Virgin Islands. Although NDs can use a variety of conventional diagnostic techniques and pharmaceutical agents, including oral anti-diabetic medications and insulin, their therapies focus on natural products, mind-body techniques, detailed nutrition counseling, physical activity prescription, and stress management recommendations delivered through individualized health promotion counseling [11]–[13]. The clinical effectiveness of ND care for diabetes has been recently explored [14]–[16] but patients’ experiences with ND care have never been investigated qualitatively.

To be effective, treatment approaches for diabetes and other complex chronic conditions need to effectively engage patients in their own care [17], [18]. In order to better characterize patients’ experiences using CAM for diabetes, and to better understand the patient experience with naturopathy, this study explored the experiences and perceptions of persons who had previously used only conventional medical care for their diabetes following a year of adjunctive naturopathic care (ANC).

## Methods

This qualitative analysis was conducted as part of an evaluation of an observational study of the effects of a course of adjunctive naturopathic care (i.e., in addition to continued medical care) on persons with sub-optimally controlled (HbA1c >7.5%) type 2 diabetes. Details of the study and the characteristics of ANC are available elsewhere [15]. In summary, in that study, participants could receive up to 8 visits to one of four naturopathic physicians (3 female and 1 male) over a one-year period, as an adjunct to continuing their usual medical care. The mean number of ANC visits actually made was 3.9+/−2.1, most of which were made within the first 6 months. None of the participants had previously received care from a naturopathic physician. All had access to naturopathic medicine as part of their current insurance benefits, although many may not have been aware of this. Following initiation of ANC, this observational study found improvements in patient-reported outcomes (e.g., glucose monitoring, diet, self-efficacy, motivation, and mood) and reductions in blood glucose that exceeded those for similar patients who do not receive ANC [15]. Approval for this study was obtained from the IRB at Group Health Cooperative.

### Selection of Participants and Recruitment

The 37 adults with diabetes who participated in the parent study and made at least one visit to an ND were eligible for this study. After completing their final outcomes assessment 12 months after entering the study, participants were invited to participate in either a focus group or an in-depth telephone interview focusing on their experiences and opinions about the naturopathic care they had received. Twenty-two (59%) agreed to participate; 17 attended one of three focus groups and 5 were interviewed by telephone. Characteristics of study participants are presented in Table 1.

**Table 1:** Demographics of participants and non-participants.

Characteristics		Participants(n = 22)	Non-participants(n = 15)	P value
Gender (Male, % )		50	40	0.16
Age (Years, mean/SD)		57.1 (7.6)	57.9 (7.5)	0.48
Highest Education (%)	High school, GED or less	9.1	12.5	0.69
	Some college, incl. technical	40.9	42.5	
	College graduate	50.0	45.0	
Ethnicity (White/Non-Hispanic %)		59.1	65.0	0.39
Annual Family Income (%)	<$60,000	45.4	38.5	0.41
	$60,000–85,000	13.6	20.5	
	>$85,000	40.9	41.0	
Years of Diabetes (mean/SD)		6.55 (3.95)	7.22 (4.19)	0.27
Baseline Hemoglobin A1c (mean/SD)		7.91 (0.58)	8.01 (0.56)	0.23
Rating of Conventional Healthcare for diabetes*	Perceived Effectiveness + (mean/SD)	1.82 (0.85)+	1.81 (0.91)^+?^	0.98
	Moderately/very satisfied with care (%)	90.9	81.2?	0.38
Rating of Naturopathic Healthcare for diabetes*	Perceived Effectiveness + (mean/SD)	2.24 (SD 0.95)+	2.86 (0.95)^+?^	0.07
	Moderately/very satisfied with care (%)	95.2	57.4?	0.01

* at 6-month follow-up,

+ 0 to 5 scale: 5 = most effective,

? n = 14 for perceived effectiveness/satisfaction items.

### Development of the Interview Guide

Three popular health behavior models describing the processes of decision-making, self-efficacy, and behavioral outcomes (Social Cognitive Theory [19], the Health Beliefs Model [20], and Self-Determination Theory [21]) informed development of our interview guide [the complete interview guide is available in Focus Group Guide S1]. The interviews explored: 1) motivations and reasons for participating; 2) general descriptions of their experience, including anything unexpected; 3) positive and negative experiences with ANC; 4) new information or insights gained from the ND experience; 5) whether and how the ND addressed health behaviors; 6) behavior changes made or contemplated as a result of the experience; and 4) comparisons of naturopathic care and conventional care for diabetes.

### Focus Groups and Interviews

Three trained qualitative researchers conducted the interviews and focus groups. Focus groups were approximately 90 minutes and interviews ranged from 25 to 60 minutes. All were tape-recorded and transcribed by a professional transcriptionist. Transcripts were imported into Atlas.ti version 6.2 (Berlin, Germany), a qualitative software that assists with coding and data management. Participants received a small incentive payment.

### Analysis

We used an inductive/deductive content analysis approach [22]. We first abstracted information pertaining to the key elements identified in the interview guide and developed an a priori code list based on the topics of inquiry. A second researcher reviewed the code list and additional codes were added based on her initial read of the transcripts; this yielded 127 unique codes. All 3 researchers then coded transcripts separately. Atlas.ti mapping features were used to aggregate codes into themes, eliminating responses that were off-topic or were isolated opinions not expressed by at least 3 participants. Redundant responses and shared opinions were aggregated into super-codes and sorted by code density as well as by participant ID (to account for repetitive statements by individual participants). The analysis team used an iterative process to discuss the themes, clarify and expand upon interpretations of findings, and contextualize the coded responses back into the transcripts of the full conversation. When disagreements were identified, the researchers reviewed the original transcripts to achieve consensus on the intent of the participants’ comments. Concepts and themes were also organized by participant ID to quantify the relative frequency of different characteristics of the care experience.

The characteristics of study participants and non-participants were compared using unpaired T-tests (Table 1). The only statistically significant difference was that participants were more likely to have been satisfied with their naturopathic care.

## Results

Our findings first describe why patients wished to try ND care and then highlight the nine major themes that emerged from the analyses. Three of these themes characterize the ND-patient *interaction* (patient-centered, holistic, and collaborative) and five characterize the *content* of the encounters (individualized and detailed health promotion, counseling that promoted self-efficacy, pragmatic and practical self-care recommendations, novel treatment options that fostered hopefulness, and patient education that addressed both diabetes self-care and general health). The final theme - contrast between ND care and conventional medical care - was often mentioned in the context the other themes but warranted description as a separate theme.

### Reasons for Participation in a Study of Naturopathic Care for Diabetes

Three major reasons were reported for participating in a study of ND care for diabetes (Table 2). Most respondents wanted to try a different approach to diabetes. About 40% expressed frustration with their current diabetes care and were concerned about progression of the disease, especially the need to increase medication or initiate insulin injections. About a third (32%) were curious about naturopathic medicine and were attracted to a more natural approach to health and disease.

**Table 2:** Reasons people with diabetes chose to participate in a study of naturopathic care (n = 22).

Reason	%*	Example quotes
**Desire for a different approach** **(not specifically CAM)**	59	“I was interested in something else. I agree with medicine up to a point – I had it coming out of my ears. I wanted something different.”
		“If it was another MD, I don’t know – you know, I have an MD. I have gone through the typical medical array of people that you go to when you have diabetes. I thought it would be a different perspective, maybe helpful.”
**Frustration with perceived ineffectiveness/** **limitations of conventional medicine**	41	“It seemed like all my doctor was pushing was medicine, get that A1c down, more medicine, more medicine, insulin. Even though I knew there were these other things, like exercise and eating better and all that, we don’t discuss that”
		“I just didn't see to be doing any good with standard medicine”
**Specific interest in trying naturopathic** **medicine or CAM**	32	“I was just curious and I always wanted to see a naturopathic doctor”
		“I prefer just to go natural with the body”
		“I wanted to reassess my lifestyle, the things I do.”

* Sums to >100% because some participants offered multiple responses.

### Themes Related to the Nature of Naturopathic Care (Table 3)

**Table 3:** Themes related to the nature of naturopathic care (n = 22).

Theme	%*	Example codes and patients’ terminology
**Patient-centered**	95	Not feeling rushed through the appointment
		Felt ND was present and focused on them during encounter.
		Feeling listened to and understood
		Empathic
**Holistic**	73	In-depth intake procedure contributed to feeling understood as a whole person
		Other health problems were addressed as influences on the management of diabetes
		Psychosocial influences on health were addressed
**Collaborative**	68	Patient preferences and input were solicited
		Feeling supported, “cheered on”
		Treatment recommendations were individualized
		Accountability was established with individualized follow-up and monitoring schedules

* Sums to >100% because some participants offered multiple responses.

#### Theme 1: Patient Centered

Various aspects of the patient-centeredness of the interaction, a major focus of CAM and integrative medicine [23], [24], were noted by 95% of participants. Participants commented that the relatively long visits with NDs (30–60 minutes) created an opportunity for a different type of interaction.

*“It’s like they [MDs] have a set amount of time allocated for each patient – maybe 15 minutes. So I have a choice between my broken finger and my diabetes. That’s what it feels like. [The ND] was present in the moment. I couldn’t tell if she was thinking of the next patient and [thinking] ‘I have to finish here so I can do the next thing’. She was very thorough too. She would look me in the eye; she was comfortable with her position. And I guess I liked the power and control she gave me over my disease.”*

*“I think the medical doctor is maybe too accustomed to seeing so many people with diseases and the progressions -- I didn't feel like the experiences or the symptoms that I was having were, I don't know, weren't maybe as worthy of treatment. He had seen people dealing with worse things than what I was dealing with. With the naturopath it was -- if it affects you, then it's real and let's take care of that.”*

Patients reflected on characteristics of the therapeutic relationship they developed with the naturopathic physicians. They used words like “casual,” “relaxed,” “personal” to describe NDs’ communication styles in contrast to using words like “formal,” “limited” – both in time and focus, and “structured” to characterize their experiences with conventional medicine.

*“I'm more at ease when I talk to my naturopath. I'm very honest with her. We have more time, and she would really listen and give me more detail about what will happen. With my primary doctor, she is wonderful too, it's just the time limit that she can talk with me. The next step would be insulin is what my primary doctor said; that's what pushed me to go into the study – I didn’t want to go see my primary doctor because I know she just goes with whatever one is saying about statistics or numbers that come out from the lab test results. It's a different atmosphere, a different way of expressing myself to the two, the primary and the naturopath.”*

*“She was very easy to talk to. I felt that she really listened and she was present with me in the moment. And that she was not holding back any information. She was genuinely forthcoming with the information that she had. There was, well, if it gets worse, we’ll try this. It’s like, let’s take these steps; and it was really kind of laid out.”*

#### Theme 2: Holistic

Patients often said they were surprised by the “whole” or “holistic” nature of ND care. Several commented that the ND attended not only to their diabetes but also to other aspects of their health and life that might impact their diabetes and their ability to engage in self-care. Several participants noted the beneficial effects of the ND’s attention to their psychoemotional state.

*“I would say that [the naturopathic] approach is dealing with your whole person, not just the specific ailment like diabetes…if you fix all these other things, everything else is going to be better. Your numbers are going to be better and so on. So she really helped resolving those by recommending other things to do.”*

*“I think one difference is, my medical doctor seems to want to manage symptoms. And the naturopath wants to get kind of to the bottom of things, take all the layers off to get to and address the root issue.”*

#### Theme 3: Collaborative

Patients reported that the NDs employed a collaborative approach that respected patient autonomy and engaged them in making decisions about following different treatment approaches to improve glycemic control. A subtheme reflected the educational component of the ND-patient interaction; patients are not only engaged in selecting the course of treatment but they are engaged as active participants in evaluating the effects of the treatment, consistent with the emerging societal prioritization of “patient centered outcomes” [25], [26].

*“It was a two-way street, not a one way street. My PCP, I love him, he is still my physician, but I felt that on a professional level, he was so squeezed…So with the naturopath, she always said what did you do? Let's look at the results. I always had the results. ‘Okay, this is okay, this needs work’, and she would always tell me something else. At the end she would always ask ‘what would you like to do?’”*

*“[The MDs] look at numbers and then prescribe a pill. I thought that was perfectly normal until [I worked with] the naturopath who said I think we can fix this with a change of diet. That got my attention. The changes were sort of like why don't you try this and so rather than what my pill doctor said, which is you will take this, this, and this. I didn't know what the ramifications were of missing it or taking too much or anything like that. It was a much different experience with the naturopath.”*

### Themes Related to the Content of Naturopathic Care (Table 4)

**Table 4:** Themes related to the content of naturopathic care (n = 22).

Theme	% *	Example codes and patients’ terminology
**Health promotion counseling: detailed** **& individualized**	100	Exercise prescriptions common and goals established with patient’s input
		Naturopathic diet recommendations given [16]
		Stress management routinely discussed
		Other lifestyle change addressed such as promoting positive mental/emotional health
**Counseling promoted patient empowerment**	95	Learning to engage in self-reflection/self-awareness
		Increased behavioral capabilities/competence
		Strategies for prioritizing self-care
		Understanding and executing self-management activities
**Recommendations were pragmatic**	82	“Tips” about diet, exercise and other health behaviors emphasized real-world implementation
		Problem-solving (barriers to behavior change, reasons for unexpected blood sugar changes) occurred
**Novel & complementary treatment options** **were offered**	82	Alternatives to pharmaceutical management (i.e. lifestyle change, dietary supplements) created hopefulness
		Feeling open to trying new things like mind-body approaches
		Trying dietary supplements & natural products for both glycemic control and other health concerns
**Information about diabetes and self-management improved health literacy**	77	Felt better educated about diabetes as a disease and goals for self-management
		Felt better educated about the role of psychosocial, behavioral, and emotional factors in relation to blood sugar
		Received educational materials from ND

* Sums to >100% because some participants offered multiple responses.

#### Theme 4: Health promotion counseling that is detailed and individualized

When describing the topics discussed with the NDs, all participants noted the emphasis on health promotion. Many participants emphasized that the diet, exercise, and stress management strategies they learned differed in quantity and specificity from those they had previously received in diabetes education classes or from their primary care provider:

*One of the things that naturopath did was explain things about lifestyle. [Other providers have] tried to talk to me about that. I did go to conferences [at the hospital] for diabetes and stuff like that. Stress does figure into it. One of the practical impacts of the stress is you don't get enough sleep. What I found is it was diet, stress, sleep. It's kind of a package; everything went together. The naturopath, even though he didn't quite explain it that way, that's what he was trying to get at. All these pieces fit together.*

*I thought it was more personal in nature and more personal directions for me to go, and here is a book on nutrition, here's a book on diabetes. And so on. It was: here's something you can do, what you need to change as far as eating goes, and exercise, and all that. More specific to me.*

Regarding the results of working with an ND on her diabetes, another participant reported:

*Well, probably some dietary changes, and paying more attention-- for some reason when you go to the dietitian, things just don't seem to come together. So trying to eat every 4 hours, that type of thing. Not that it always works. I probably eat too much. But I'm paying more attention to the diet. The times [I eat]. What's in the diet. Like proteins for sure, and trying to eat more vegetables and fruits and stuff. I joined a gym, which I thought I would never do.*

#### Theme 5: Counseling promoted patient empowerment

Participant reports suggested that the naturopathic counseling style helped them increase their locus on control, with numerous patients reporting experiencing new “confidence,” feeling “less overwhelmed,” and feeling “in control.”

*“I was surprised at how much it depended on me. You know, I guess this process has shown me how much my health is in my hands. That I’m not having to give it over to an MD to watch me deteriorate, and that certain markers mean certain things because this is the way the disease progresses. [The ND] was more like, take these steps and these things can change. Keep you as healthy as we can, as long as we can.”*

*“My naturopath was very encouraging. She would encourage me to take the power and control she was trying to give me. And I would say that was for the best. I felt very encouraged to take control.”*

*“For me it [diabetes] is less of an obstacle. It's more something I can deal with and work with. Yes, I will fall off the wagon periodically, but it won't be the end of the world if I do. He [the naturopath] helped me stop making it something that wasn't … a huge obstacle, something to get stressed out about.”*

#### Theme 6: Recommendations were pragmatic

The third most common topic was the practical nature of the suggestions about self-management and implementing the health promoting behavioral changes. Participants described the advice they received as “specific tips,” “practical for real-life,” and focused on “problem-solving” experiences like elevated blood sugar readings and barriers to self-care. Patients felt these “tips” influenced their confidence in self-care by making changes seem less overwhelming and more feasible.

*“It felt like she had made a point of showing me all the little tricks that you generally overlook, because no one ever taught them to us. Just taking a small piece of protein at bedtime will bring your morning numbers down by 10 or 25 points; I never heard that. Or like a teaspoon of cinnamon will help your numbers. And I tried that and it worked. She had all these things.”*

*“I got specifics from the naturopath. You know, it’s hard for me to fit exercise into my daily activities. I would fall down on that and beat myself up on it. And then I wouldn’t want to tell [the ND] exactly what I have done. She helped me decide on walking as a regular program and when I couldn’t get there, she did follow up with me. She suggested I do the minimum I can do and that will be fine to start. Don’t beat myself up about it. So that was different from that I would have gotten from my GP.”*

#### Theme 7: Novel and complementary treatment options were offered

Another topic of the clinical interaction reported by participants was exposure to new therapeutic options. These new options appeared to give patients a new sense of hopefulness or optimism about their diabetes, both in terms of self-management and in an expanded view of their medical team.

*He prescribed supplements. And I already have a regimen of medication that I take -- and supplements -- you know, just the general multivitamin minerals. But they weren't specific to diabetes. [The ND recommended ones that] were more specific. And they made such a difference that -- it was more a realization that when I ran out and I had to wait a week for payday and going back to the way it was, knowing that that supplement had created such difference was kind of surprising to me.*

*I was complaining about not having time, and she said but you have a Wii, the game machine. She said play the Wii, and if you are doing the boxing thing or you are doing any of those things, it's exercise. She showed me that there are alternatives to getting on a treadmill or going to a gym. It's accessible whenever you want it. The weather doesn't have to be nice. That for me was really helpful, alternative ways of looking at things and doing things.*

#### Theme 8: Information about diabetes and self-management improved health literacy

Patients reported that they learned useful new information from the NDs. Additional time spent on fundamental health education appeared to help participants develop a better understanding of their chronic disease, evaluate a variety of treatment options, and make informed decisions regarding their self-care and treatment options.

*I’m not a big supplement guy and so she was talking about fiber. Fiber does this and that – [the ND] had a fairly convincing argument of why that was important and what it could do for me. And so I was willing to try it. The [medical] doctor said: yeah, fiber is important but never really got the full explanation. So now I get my 100% daily requirement of fiber everyday and then some. I believe it’s had some effect on my health.*

*She gave me 4 different things to try. [The ND said] well then, let's play with fruit. So we had berries with sugar or coffee sweetener. And the cream. You could take berries, cream with no sugar, and Splenda, that reduces my sugars 20 to 50 points in the morning. And we played with that. And we tried apples. And we had a different experiment going for 3 weeks to find out what works with my body.*

#### Theme 9: Contrast between naturopathic care and conventional medicine

As illustrated by many of the quotes included for the themes described above, nearly all participants contrasted their NM experience with conventional medicine. The attributes that stood out as most different included the focus on psychosocial factors, especially stress, as contributors to glycemic control; the use of dietary supplements and natural products; and the emphasis on education and developing an understanding of self and health. Participants also commented about the greater emphasis by NDs on individualized goals (e.g. starting with a walking program or minimal exercise and building upon it).

Additionally, participants reported the benefits of having both naturopathic and conventional providers on their care team. Many reported both approaches made useful contributions to their overall diabetes management, with some reporting their experience with the ND helped them re-engage with their conventional primary care providers and/or improve their adherence to prescription medication:

*“You’ve got to know about yourself and know what your numbers are and ask questions and force them to pay attention…I’m going to go back to my GP and ask him “Are you in the driver’s seat? Are you going to help me?”*

*“I was concerned that I could become dependent on insulin. [The ND] was encouraging: that in most cases, if you lose weight, then your dependency on insulin also goes down. So not to be afraid of taking insulin. She was actually the one that got me taking the insulin regularly in the first place.”*

The only complaint about their naturopathic care experience was the high costs of recommended dietary supplements, which were not covered by insurance.

## Discussion

This study is unique in that it captures the opinions of naturopathic-naïve patients who were exposed to ND care in a pragmatic trial conducted in a community-based setting. The percentage of patients with diabetes across the U.S. using naturopathic medicine as a specific form of CAM is unknown, but among naturopathic medicine users, 16.8% have diabetes which is almost twice the national prevalence of diabetes [11], [27]. The quotations reported here provide insight into what patients’ might experience when working with naturopathic physicians to address diabetes and improve glycemic control.

Aspects of both the nature and the content of ND care emerged as important themes in patients’ perceptions of their first experiences with naturopathic care. Many of these perceptions contrasted ND care with MD care, highlighting key distinctions that may have important implications for understanding how care for diabetes might be improved.

The characteristics of the nature and content of naturopathic medicine reflect features of care that are well-supported by the literature as necessary for effective behavior change counseling, including being intensive [28]–[30], multi-factorial [31]–[34], collaborative [35], [36], and targeted to the patient and their individual needs [31], [37]. There were many examples of how patients increased self-efficacy during their experience working with an ND; 95% reported experiencing a sense of empowerment. Examples of self-efficacy changes reflected in the narrative include “it’s up to me,” “I feel more in control now,” “I can succeed in managing it.”

The characteristics of care delivered by naturopathic physicians appear congruent with domains of health promotion theory known to be important to effect behavior change. For example, according to Social Cognitive Theory [19], to change behaviors, people must have the confidence, or *self-efficacy*, to persist even when faced with obstacles. If they do not feel that they can exercise control over their health behavior, they will not be motivated to act and behavior does not change.

Other domains of the Social Cognitive Theory (SCT) of health behavior change are also reflected in patients’ experiences with NM. For example, the “tips” and strategies patients learned from NDs contributed to their *Behavioral Capability*–their knowledge and skill in mastering healthy behaviors. Patients described how they had been engaged in *Observational Learning* by experimenting with different dietary strategies to see how their blood sugar control would be affected. *Reciprocal Determinism,* which refers to the dynamic interaction between the person, their environment and the behavior, was reflected in participant’s comments about attention to psychosocial barriers and the holistic approach that addressed more than blood sugar control. *Outcome Expectations,* the final domain of the SCT, may have been clarified by the ND emphasis on health education and individualized goal-setting. Future research should further examine if the congruence between characteristics of ANC with conceptual models of health behavior change can be quantified. If so, the findings may be useful logic models for designing health promotion interventions to improve diabetes self-care.

Patients reported they had a unique experience working with the naturopathic physicians, even though they had diabetes for an average of seven years and were receiving care in a managed healthcare delivery system known for its high quality care, including access to diabetes education programs and nutritionists. Many reported that they loved their MDs, but may not have realized the limitations of medical care until they experienced something different. Previous reports have emphasized the similarities in ND and MD care delivery, but these studies have been largely quantitative (i.e. types of diagnoses, categorical similarities in visit content such as history taking, lab ordering, prescribing) [12], [38]. An obvious difference between ND and MD care is the average visit duration (about 40 minutes for NDs [12] and 21 minutes for primary care MDs [39], although within the healthcare system studied, primary care visits are allocated 20–30 minutes per patient. While longer visits do appear to reduce laboratory orders and prescribing, studies have not found improved care in diabetes or other chronic conditions simply by allowing for more time [40], [41] suggesting the approach and content of the ANC visits set this experience apart, rather than time *per se*.

The findings of this small exploratory study are limited by the small number of NDs (3 female and 1 male) involved and restriction to patients in an integrated health care delivery system in Western Washington. Enrollees in this health system are known to have more years of education than the national average [42]. In addition, although the 22 participants in this qualitative study were not significantly different than the 15 who declined to participate on a variety of sociodemographic and diabetes-related variables, they had been more satisfied with their experience of naturopathic care. As a result, the views of the participants described in this study are almost certainly more positive than would have been the case had we had a higher participation rate. However, given that 48% of patients with sub-optimally controlled diabetes in our target population said they would be very likely to try ND care for their diabetes if covered by their health plan, there appears to be a large pool of individuals interested in and open to complementary approaches to care of their diabetes [43].

As our current healthcare system struggles to meet the needs of patients with chronic diseases such as diabetes, effective strategies are needed to help patients gain self-efficacy to engage in health promoting behaviors. Based on the results reported here, the utilization of naturopathic physicians as an adjunct (in addition) to conventional primary care has benefit beyond primary care alone, however, future research is needed to confirm these benefits and if confirmed, elucidate whether the benefits observed are additive, substitutive, or synergistic to usual care. Care delivered by naturopathic physicians appears to be a useful model that has some preliminary evidence from observational studies of beneficial effects on patient behaviors and clinical outcomes [15]. Additional research using a randomized clinical trial design should test the utility of this unique approach in larger, most diverse populations. If more rigorous studies show that adjunctive naturopathic care provides substantial benefits beyond those of medical care alone for people with diabetes, new models of integrative care will warrant serious exploration.

## Supporting Information

Focus Group Guide S1. The full text of the focus group interview guide.
(DOC)
