# Supplementary material for: Patient-Reported Experiences with First-Time Naturopathic Care for Type 2 Diabetes
Source: PLoS One. 2012 Nov 7;7(11):e48549. doi: 10.1371/journal.pone.0048549 (PMC3492455; doi:10.1371/journal.pone.0048549)
Supplement: Focus Group Guide S1. — The full text of the focus group interview guide. (DOC) [file pone.0048549.s001.doc]

# S1. Focus Group Guide

# Experience with Naturopathic Medical Care for Diabetes

# Focus Group Guide

# 08-11-10 Version

# Thank you so much for coming today to share your ideas and experiences with us. We are interested in hearing about your experience seeing a naturopathic doctor as a part of this study and how it impacted your opinions on your health and healthcare. This information will help us more fully understand the potential value of naturopathic care for persons with diabetes.

# There are no “right” or “wrong” answers, so please feel free to share whatever ideas and opinions you like as I ask the questions. You do not have to answer any question that you do not want to answer. We will be recording our conversation today and the study team will only hear the recordings. To protect privacy, we ask that you only use first names around this table and try not refer to your doctors by name. If you forget and say their name, it’s ok, we’ll block it out of the transcript.

# Does anyone have any questions? I would be happy to answer them now or at any time during the discussion.

1. We’d like to start by going around the table having each of you tell us 1) your **name,** 2) **one fact about you** that you think will help us have a better sense of who you are, and 3) whether, prior to the BAND study, you had **ever been to see a naturopath** or any other type of alternative health care provider. If you had, we would like you to briefly describe that experience for us.
2. When you signed up to participate in this study, how many of you were aware that you might receive care from a naturopath? To what extent did the opportunity to receive naturopathic care motivate you to participate in the study? For those of you that weren’t aware that this would be part of the study, what were your first thoughts when you were told that you would be receiving care from a naturopathic physician?
3. What **surprised** you most about the care you received from the naturopathic physician?
4. In your opinion what were the most notable **differences between the care** you received from the naturopathic physician and the care you tend to receive from Group Health physicians?
5. What did you think was **similar between the care** you received from the naturopathic physician and the care you receive from Group Health physicians?
6. What did you **like most** about your experience with the naturopathic physician?
7. What, if anything, didn’t you like about your experience with the naturopathic physician?
8. What new **information or ideas** did you learn about your body or your health from the naturopathic physician?
9. What were some of the things your naturopathic physician told you that were new to you?
10. What are some of the **recommendations** the naturopathic physician made regarding taking care of your **overall health**?
11. What **recommendations** did the naturopathic physician make regarding your **diabetes**?
12. Now I’d like to take a few minutes to go around and have each of **you briefly describe your experience with the naturopathic physician** especially with regard to the recommendations they made for you and whether you followed through with those recommendations.
13. Some **common themes** I heard about your experiences were <summarize>. In listening to other people’s experiences were there other things that you felt were similar to your own experiences and/or feelings?
14. Overall, are you going to **do anything differently** based on the information and recommendations you received from the naturopath?
    - How has the experience changed the way you think about your health?
    - How has the experience changed the way you think about your diabetes?
    - Has your ability or willingness to follow your Group Health doctor’s recommendations changed?
15. How many of you will **continue** to work with a naturopathic doctor in the future?
    - Why or why not?
16. How many of you would **recommend a naturopathic physician to a friend**? Why or why not?
    - Those of you who would recommend a naturopathic physician, what would you say to your friend about why they should go? What are the benefits you would highlight?
17. Is there anything else you feel it would be helpful for us to know?

Thank you so much for sharing your ideas with us. This has been really helpful.
